# Rationale, design and baseline results of the Treatment Optimisation in Primary care of Heart failure in the Utrecht region (TOPHU) study: a cluster randomised controlled trial

**DOI:** 10.1186/s12875-015-0347-1

**Published:** 2015-10-07

**Authors:** Mark J. Valk, Arno W. Hoes, Arend Mosterd, Marcel A. Landman, Berna D. L. Broekhuizen, Frans H. Rutten

**Affiliations:** Julius Center for Health Sciences and Primary Care, University Medical Center Utrecht, Stratenum 6.131, PO Box 85500, 3508 AB Utrecht, The Netherlands; Department of Cardiology, Meander Medical Center, Amersfoort, The Netherlands; van Hardenbroeklaan 21, 3832 CK Leusden, The Netherlands

**Keywords:** Heart failure, General practice, Drug treatment, Training, Cluster randomised controlled trial

## Abstract

**Background:**

Heart failure (HF) is mainly detected and managed in primary care, but the care is considered suboptimal. We present the rationale, design and baseline results of the Treatment Optimisation in Primary care of Heart failure in the Utrecht region (TOPHU) study. In this study we assess the effect of a single training of GPs in the pharmacological management of patients with HF.

**Methods/design:**

A cluster randomised controlled trial. Thirty primary care practices are randomly assigned to care as usual or intervention defined as a single training in the up-titration and management of HF drug therapy according to the heart failure guidelines of the European Society of Cardiology (ESC). Patients with a GP’s diagnosis of HF will be re-evaluated by an expert panel of two cardiologists and a GP with expertise in HF to come to a definite diagnosis of HF according to the ESC heart failure guidelines. Those with definite HF will be analysed in this study. Drug use will be measured after six months, health status after twelve months, and heart-related hospital admissions and all-cause mortality after two years.

**Discussion:**

Our cluster randomised trial will show whether a single training of GPs improves the pharmacological management of patients with HF and confers beneficial effects on health status after one year, and cardiac hospital admissions and all-cause mortality after two years of follow-up.

**Trial registration:**

ClinicalTrials.gov Identifier NCT01662323

## Background

Heart failure (HF) is an important medical and health care problem with great impact on patient’s health status and life expectancy. The initial diagnosis of HF is mainly made in primary care, and is still often based on the clinical assessment only, irrespective of the general knowledge that such a diagnosis solely based on clinical grounds, without echocardiography bares the risk of both overdiagnosis and underdiagnosis, certainly in the early stages of slow-onset HF [1–4]. This knowledge is also important because a previous study in primary care suggested that uncertainty regarding the diagnosis of heart failure is an important barrier to adequate drug treatment by GPs [5]. Moreover, echocardiography is needed to differentiate HF with a preserved ejection fraction (HFpEF) from HF with a reduced ejection fraction (HFrEF) [1]. Although, both types are part of the heart failure spectrum, the treatment is different. In HFpEF it is focussed on i) release of symptoms with diuretics in case of fluid retention, ii) adequate blood pressure control, and iii) management of comorbidities. Importantly, however, none of these treatments has a clear prognostic benefit and thus lacks a real evidence-base. On the other hand, for HFrEF there are multiple drugs including angiotensin converting enzyme inhibitors (ACE-i) or angiotensin receptor blockers (ARB), β-blockers, and mineralocorticoid receptor antagonists (MRA), and devices that reduce mortality and heart failure hospitalization as it does improve quality of life [1]. Ivabradine should be considered in the subgroup of patients who remain symptomatic with the three aforementioned drugs, and have sinus rhythm with a pulse frequency higher than 70 beats/min in rest [1].

**Table 1:** The diagnosis of heart failure according to the ESC guidelines on heart failure 2012 [1]

Diagnosis of HF with a reduced ejection fraction (HFrEF)	Diagnosis of HF with a preserved ejection fraction (HFpEF)
Symptoms typical of HF	Symptoms typical of HF
Signs typical of HF^a^	Signs typical of HF^a^
Reduced left ventricular ejection fraction	Normal or only mildly reduced left ventricular ejection fraction and left ventricle not dilated
	Relevant structural heart disease (LV hypertrophy/left atrial enlargement) and/or diastolic dysfunction

*HF* heart failure

^a^Signs may not be present in the early stages of heart failure (especially in HFpEF) and in patients treated with diuretics

Previous studies showed that general practitioners (GPs) are less successful than cardiologists in up-titrating HF drugs according to guidelines [2, 3, 6–9]. Nevertheless, they adequately maintain the drug management initiated in secondary care, as good as done in heart failure clinics [10, 11].

We aim to determine whether a single training of GPs focused on the drug management improves the pharmacological management of patients with definite HF, HFrEF and HFpEF seperately. Additionally, we determine if it has a beneficial effect on health status, cardiac hospital admissions, and all-cause mortality.

### Key objectives

- To assess how many patients labelled with HF in primary care really have heart failure.
- To assess the effect of a single half-day training on drug management in heart failure on drug use, health related quality of life, heart failure hospitalizations, and all-cause mortality after six months, 12 months, and 24 months, respectively.

## Methods

### Study design

**Fig. 1:**
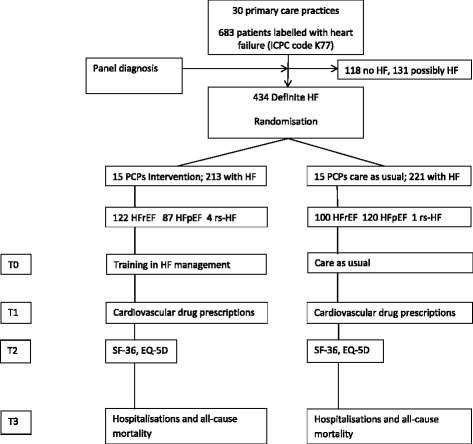
Study scheme. ICPC = International Classification in Primary Care, PCP = primary care practice; HFrEF = heart failure with reduced ejection fraction, HFpEF = heart failure with preserved ejection fraction, rs-HF = isolated right-sided heart failure. SF-36 and EQ-5D are health related quality of live questionnaires. T0: start of the study with training of the GPs, GP trainees and practice nurses of the intervention group. T1: Six months of follow-up; assessment of cardiovascular drug use in both groups in comparison to baseline. T2: Twelve months of follow-up; questionnaires on health status (SF-36 and EQ-5D will be filled out by participants in both groups. T3: Two years of follow-up; assessment of hospitalisations and all-cause mortality in the electronic medical files of the GPs in both groups

We designed a cluster randomised trial with randomisation at the level of the primary care practices, to help prevent contamination with the intervention. Thirty practices are randomly divided into two groups of fifteen. The intervention group will receive a half-day training on HF management and will receive an up-titration chart for daily use in the management of HF patients during the study, while the control group will not receive specific training, and provides care as usual. The study starts with the training, and participants in both groups will be followed up for two years. After six months of follow-up the electronic medical files will be scrutinized for (change in) prescriptions of drugs in comparison to baseline. Twelve months after the training the participants will be sent a questionnaire on health status. After two years, hospital admissions and all-cause mortality will be assessed, by again scrutinizing the GPs’ electronical files (Fig. 1).

**Fig. 2:**
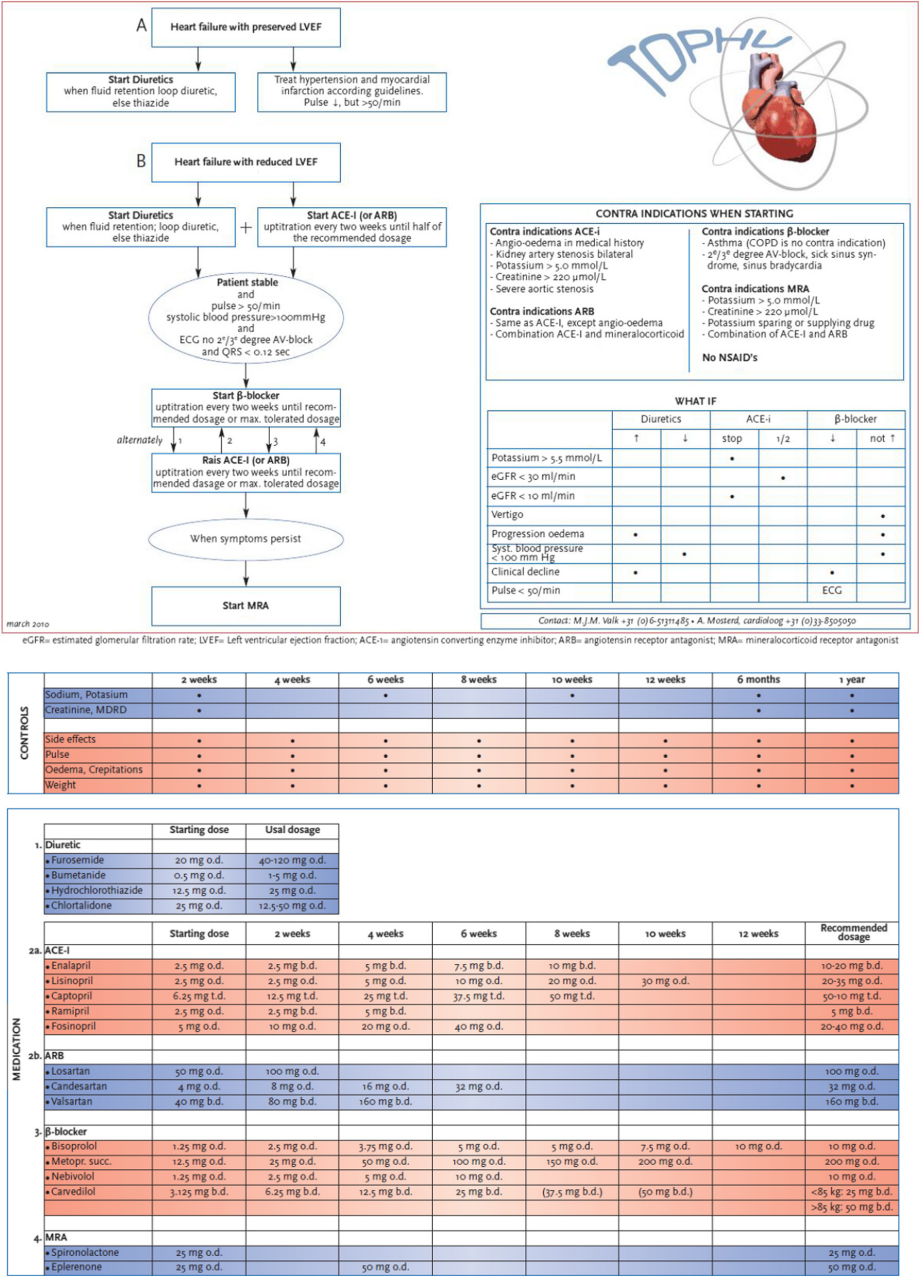
Up-titration scheme as provided on a leaflet to be used in everyday practice by the GPS in the intervention group

### Recruitment of general practitioners

General practitioners (GPs) will be recruited in and around Amersfoort, a city in the centre of the Netherlands. A representative group of 195 GPs working in group, duo or solo practices in urban, suburban, and rural areas were invited by letter. Forty-five GPs working in 30 GP practices consented to participate. They are all familiar with the Dutch GP guideline on heart failure [12]. The participating general practices were randomly allocated to either the intervention or care as usual group. The project manager undertook randomisation in a blinded fashion. GPs working in one practice were allocated to the same group, to avoid contamination of GPs and participants between the two groups, which can occur if randomization is performed at an individual participant level.

### Study population and recruitment

All citizens in the Netherlands are registered with a GP, also those who receive cooperative care from a medical specialist, except those living in a nursing home or hospice. All patients enlisted with the participating GPs and who have a GP’s diagnosis of HF encoded according the International Classification of Primary Care (ICPC) code K77 will be assessed if this ICPC code was allocated at least twice for patient contacts, to prevent single accidental miscoding. Five months before the start of the study, the electronic medical files of the 30 participating GPs will be scrutinised for such patients labelled with ICPC code K77, and if echocardiographic results are missing, the GPs of both groups will be urged to consider referral for echocardiography. The start of the study is the date of training of the GPs in the intervention group. An expert panel consisting of two cardiologists and an experienced general practitioner will evaluate all available diagnostic information from the electronic medical files of the GPs, including echocardiography results mentioned in cardiologist’s papers when this investigation was performed. They decide whom of those with ICPC code K77 has definite HF, probably or possibly HF, or no HF according to the European Society of Cardiology guidelines [1]. Only patients who have definite HF according to the panel will be analysed in the cluster randomised trial. All participants will be asked to give written informed consent.

### Sample size

We base our sample size calculation on the cases with definite HFrEF. We speculate that 30 % of them will be on a β-blocker and 60 % on an angiotensin converting enzyme inhibitor (ACE-i) or angiotensin receptor blocker (ARB) at baseline, and that after six months of follow-up after the training session these percentages will have increased to 60 and 90 %, respectively in the intervention group, while remaining the same in the control group. To prove a difference of 30 % in prescription rates in ACE-i/ARBs and β-blockers between the intervention and care as usual arm after six months with an alpha of 0.05, a power of 0.80, and an intra-cluster correlation coefficient of 0.05 [12], and a cluster size of 5, we need 47 patients with HFrEF in each study arm. Considering a drop-out of 10 % we aim to include 52 participants in each arm (total 104). We calculated that around 30 general practices should participate to recruit 104 patients with definite HFrEF.

### Intervention and care as usual

GPs, GP trainees, and nurse practitioners of general practices of the intervention arm receive a two hours lasting interactive training on the diagnosis and pharmacological management of HF by a cardiologist and GP with expertise in HF. Special attention will be paid to initiation and up-titration to optimal dosage of evidence-based drugs in patients with HFrEF, especially in the drugs that should always be considered to be prescribed; ACE-inhibitors or ARBs, beta-blockers, and MRAs. The ‘hand-out’ leaflet to be used in everyday practice will be explained (Fig. 2). This leaflet provides detailed information on the intervals in the up-titration, what should be checked at control visits, contra-indications of the cardiovascular drugs, and laboratory tests needed (i.e., creatinine and potassium levels). Differences in the drug management of patients with HFrEF and HFpEF will be explained, as also the most common interaction and adverse effects of HF drugs. Finally, general aspects such as adherence, and polypharmacy and options of self-care will be discussed interactively. Participants of the training will not be reinforced by reminders, newsletters, or other communications after the training. GPs, GP trainees and practice nurses in the care as usual group will not receive such a training nor an up-titration chart.

### Panel procedure and the definite diagnosis of heart failure

The expert panel will consist of two cardiologists and a GP experienced in HF. They will decide during consensus meetings on the presence or absence of HF following the criteria of the HF guidelines of the ESC (Table 1). In addition to symptoms and signs suggestive of HF additional evidence from echocardiography of structural or functional abnormality of the heart at rest is needed to establish the presence of HF [1]. With the assumption that all patients labelled with a GPs diagnosis of heart failure have symptoms and signs suggestive of HF, the panel will evaluate, when available, the results from additional diagnostic testing such as natriuretic peptide values, chest X-ray, electrocardiography and echocardiography. Based on consensus, the panel decides if a patient has no HF, probably or possibly HF, or definite HF. Only patients with definite HF according to the panel will be analysed in the cluster randomized trial.

Cases with definite HF will further be subdivided in HFrEF, HFpEF, and isolated right-sided HF (rs-HF). For HFrEF, a reduced left ventricular ejection fraction (LVEF) is needed, arbitrary ≤ 45 %. For HFpEF, the LVEF should be normal or nearly normal, arbitrary >45 %, this in the presence of at least two structural or functional abnormalities related to relaxation such as a left atrium volume indexed (LAVI) >34 ml/m^2^, E/e’ >15, E/A <0.75, and/or a left ventricular wall thickness >11 mm. In those with atrial fibrillation, a LAVI >34 ml/m^2^ is sufficient for the diagnosis of diastolic dysfunction. For isolated right-sided heart failure, the LVEF should be >45 %, and the calculated peak pulmonary pressure >40 mmHg that is insufficiently explained by left ventricular dysfunction.

### Data collection

At baseline, the following data will be extracted from the electronic medical files of the participants: age, gender, cardiovascular drug use, comorbidities, the most recent blood test results including natriuretic peptide measurements (NTproBNP or BNP) and the eGFR, whether echocardiography was performed, and if the patient received cooperative care from a cardiologist. Such cooperative care is considered present when a patient consulted a cardiologist at least once in the 18 months before the start of the study. Six months after the training, the prescription of cardiovascular drugs in both arms will again be extracted from the GPs’ electronic medical files. After one year, participants in both arms will be asked to fill out two health status questionnaires (the Short Form 36 and the five dimensional Euro Qual (EQ-5D) [13, 14, 15]. Two years after the start of the study, the GPs’ electronic medical files will be scrutinized again to assess hospital admissions and all-cause mortality. See also Fig. 1.

### Outcomes

Study outcomes are the proportions of patients labelled with ICPC K77 who really have heart failure according to the expert panel, and the proportion of patients with definite heart failure and a reduced ejection fraction that received the most relevant and universally needed HF drugs, including ACE-i/ARBs, β-blockers, and mineralocorticoid receptor antagonists. Drug use at baseline and after six months will be compared between the two groups.

Health status will be assessed with the SF-36 and the EQ-5D in all with definite HF (HFrEF and HFpEF). The SF-36 is subdivided into eight domains: physical functioning, social functioning, limitations in usual role activities due to physical problems, limitations in usual role activities due to emotional problems, bodily pain, general vitality health, general mental health, general health perception. Scores range from 0 to 100. The EQ-5D questionnaire has five dimensions: mobility, self-care, usual activities, pain/discomfort, and anxiety/depression, which are divided into three degrees of severity; “no problem”, “some problems” or “major problems”. A single index score can be produced using information from these five dimensions. Higher scores on both questionnaires are associated with a better health-related quality of life.

Also cardiac and other hospitalisation will be assessed in all with definite HF, and the duration of hospitalisation.

### Data analyses

We will calculate with its 95 % confidence interval how many patients with an ICPC code K77 were correctly diagnosed with heart failure according to the expert panel.

The proportion of prescribed HF drugs between the two groups will be compared after six months taking into account baseline differences. The difference in health status between participants with definite HF in the two study arms at 12 months will be compared with ANCOVA. Differences between participants of the two groups regarding hospitalisations and all-cause mortality will be assessed after two years. A multilevel approach will be used in the analyses to correct for the fact that we randomized at the GP practice and not at the patient level.

### Regulation statement

This study is conducted according to the principles of the current version of the declaration of Helsinki and in accordance with the Dutch law on Medical Research involving Human Subjects Act (WMO).

### Ethics committee approval

The study was approved by the Regional Medical Ethics Committee (VCMO) of the Meander Medical Centre, Amersfoort, the Netherlands.

## Discussion

In this study we will quantify how many patients with a GP’s diagnosis of heart failure really have heart failure. In a randomised trial we will quantify the effect on drug use, health status and prognosis with hospital admissions, and all-cause mortality of a single training of GP’s that is focused on the drug management of patients with definite HF, and for HFrEF and HFpEF seperately.

There are several limitations to be mentioned. First, a two-hour training is short to adequately train GPs how to initiate and up-titrate heart failure medication in patients with HFrEF, even if they are familiar with heart failure guidelines, receive a helpful leaflet, and with over half of the patients receiving cooperative care from the cardiologist. For logistical and practical reasons we choose for such a single intervention because it resembles most closely post-graduate education GPs receive in the Netherlands. Secondly, we measure outcomes only once; drug prescriptions after 6 months, hr-QoL after 12 months, and CV morbidity and mortality after 24 months. More frequent measurements of outcomes would result in ‘disturbing’ GPs, and multiple times filling out questionnaires by participants. It would easily result in ‘drifting away’ from ‘real’ care as usual of those in that arm of the study. Thirdly, not all patients will undergo echocardiography in this practice-based study. The advantage of a practice study is the inclusion of ‘real’ patients and the assessment of drugs in ‘real’ practice. The downside is missings on some variables. In real live practice not everybody labeled with heart failure underwent echocardiography. Nevertheless, for our cluster randomized trial we will selectively analyse those with definite HF, that is, symptoms of HF and functional/ structural abnormalities with echocardiography, evaluated by an expert panel of two cardiologists and a GP. Fourthly, we evaluated both HFrEF and HFpEF patients, although, for HFpEF ‘clear’ evidence-based treatment is lacking. Nevertheless, HFpEF is part of the heart failure spectrum, and has nearly as poor a prognosis as HFrEF. Moreover, these patients suffer of symptoms, notably fluid retention causing shortness of breath and peripheral oedema. These symptoms can adequately managed with diuretics. Physicians should realize that symptom relieve is of utmost importance in these patients by titrating the dose of diuretics as optimally as possible. Adjustments of diuretic dose to filling status is really the ‘art’ of medicine. Even more can be done in patients with HFpEF; blood pressure and comorbidities should be adequately managed according to the ESC guidelines 2012. Finally, the recommendation to GPs in both trial arms to refer for echocardiography before the training may increase awareness of HF diagnosis and management and may dilute the effect of the intervention. 

We realize there are other options to improve the care of patients with HF in general practice, such as a multidisciplinary approach, practice nurse-led disease management, or tele-health. Tele-health, providing daily-wise data of body weight, blood pressure, pulse, and sometimes even much more biological data could also improve the care of the complex patients with HF. Many previous studies evaluated patients under the care of HF outpatient clinics receiving multidisciplinary care, and this resulted in prognostic beneficial effects [16]. Also practice nurses in primary care could be helpful in the care of patients with HF in the home setting. In the primary care setting in the Netherlands, disease-specific care pathways have been developed for diabetes mellitus, chronic obstructive pulmonary disease (COPD), and cardiovascular risk management. In these programs, practice nurses play an important role. They receive a special training to monitor these patients. These nurses, however, are not yet trained to care for HF, a disease with multiple systemic effects, and high morbidity and mortality. Training them in HF would also be an option to upgrade the care of HF in the primary care setting.

Our study approach is focussed on a single intervention that could improve care and would be easily implemented if effective. We want to improve all aspects of drug use in heart failure, also considering interaction, contraindication, and adherence. We realize that our strategy could gain by paying even more attention to self-care of patients, and by facilitating cooperative care of the cardiologist and HF nurse.

We realize that our intervention is relatively small, but importantly, we focus on probably the most important aspect of HF management, namely real adequate drug use. The advantage of our approach is that it can easily be implemented in everyday primary care.

## Abbreviations

ACE-I: Angiotensin converting enzyme inhibitors
ARB: Angiotensin receptor blockers
BNP: Brain natriuretic peptide
eGFR: Estimated glomerular filtration rate
ESC: European Society of Cardiology
EQ-5D: EuroQoL Quality of Life Scale 5 dimensions
GP: General practitioner
HF: Heart failure
HFpEF: Heart Failure with preserved Ejection Fraction
HFrEF: Heart Failure with reduced Ejection Fraction
ICPC: International Classification in Primary Care
LAVI: Left atrium volume indexed
LVEF: Left ventricle ejection fraction
MRA: Mineralocorticoid receptor antagonist
NTproBNP: N-terminal pro brain natriuretic peptide
rs-HF: Right sided heart failure
SF-36: 36-Item Short Form Health Survey

## References

[1] McMurray JJ, Adamopoulos S, Anker SD, Auricchio A, Bohm M, Dickstein K (2012). ESC guidelines for the diagnosis and treatment of acute and chronic heart failure 2012: the task force for the diagnosis and treatment of acute and chronic heart failure 2012 of the european society of cardiology. Developed in collaboration with the Heart Failure Association (HFA) of the ESC. Eur Heart J.

[2] Rutten FH, Grobbee DE, Hoes AW (2003). Differences between general practitioners and cardiologists in diagnosis and management of heart failure: a survey in every-day practice. Eur J Heart Fail.

[3] Cleland JG, Cohen-Solal A, Aguilar JC, Dietz R, Eastaugh J, Follath F (2002). Management of heart failure in primary care (the IMPROVEMENT of Heart Failure Programme): an international survey. Lancet.

[4] Remme WJ, McMurray JJ, Hobbs FD, Cohen-Solal A, Lopez-Sendon J, Boccanelli A (2008). Awareness and perception of heart failure among European cardiologists, internists, geriatricians, and primary care physicians. Eur Heart J.

[5] Fuat A, Hungin AP, Murphy JJ (2003). Barriers to accurate diagnosis and effective management of heart failure in primary care: qualitative study. BMJ.

[6] Cleland JG, Swedberg K, Cohen-Solal A, Cosin-Aguilar J, Dietz R, Follath F (2000). The Euro Heart Failure Survey of the EUROHEART survey programme. A survey on the quality of care among patients with heart failure in Europe. The Study Group on Diagnosis of the Working Group on Heart Failure of the European Society of Cardiology. The Medicines Evaluation Group Centre for Health Economics University of York. Eur J Heart Fail.

[7] Allen LA, Magid DJ, Zeng C, Peterson PN, Clarke CL, Shetterly S (2012). Patterns of beta-blocker intensification in ambulatory heart failure patients and short-term association with hospitalization. BMC Cardiovasc Disord.

[8] Ansari M, Shlipak MG, Heidenreich PA, Van OD, Pohl EC, Browner WS (2003). Improving guideline adherence: a randomized trial evaluating strategies to increase beta-blocker use in heart failure. Circulation.

[9] Muntwyler J, Cohen-Solal A, Freemantle N, Eastaugh J, Cleland JG, Follath F (2004). Relation of sex, age and concomitant diseases to drug prescription for heart failure in primary care in Europe. Eur J Heart Fail.

[10] Schou M, Gustafsson F, Videbaek L, Tuxen C, Keller N, Handberg J (2013). Extended heart failure clinic follow-up in low-risk patients: a randomized clinical trial (NorthStar). Eur Heart J.

[11] Luttik ML, Jaarsma T, van Geel PP, Brons M, Hillege HL, Hoes AW (2014). Long-term follow-up in optimally treated and stable heart failure patients: primary care vs. heart failure clinic. Results of the COACH-2 study. Eur J Heart Fail.

[12] Hoes AW, Voors AA, Rutten FH, van Lieshout J, Janssen PGH, Walma EP (2010). The Dutch College of General Practitioners guideline on heart failure (In Dutch). Huisarts Wet.

[13] Campbell MJ (2000). Cluster randomized trials in general (family) practice. Stat Methods Res.

[14] Aaronson NK, Muller M, Cohen PD, Essink-Bot ML, Fekkes M, Sanderman R (1998). Translation, validation, and norming of the Dutch language version of the SF-36 Health Survey in community and chronic disease populations. J Clin Epidemiol.

[15] Brooks RG, Jendteg S, Lindgren B, Persson U, Bjork S (1991). EuroQol: health-related quality of life measurement. Results of the Swedish questionnaire exercise. Health Policy.

[16] McAlister FA, Lawson FM, Teo KK, Armstrong PW (2001). A systematic review of randomized trials of disease management programs in heart failure. Am J Med.

